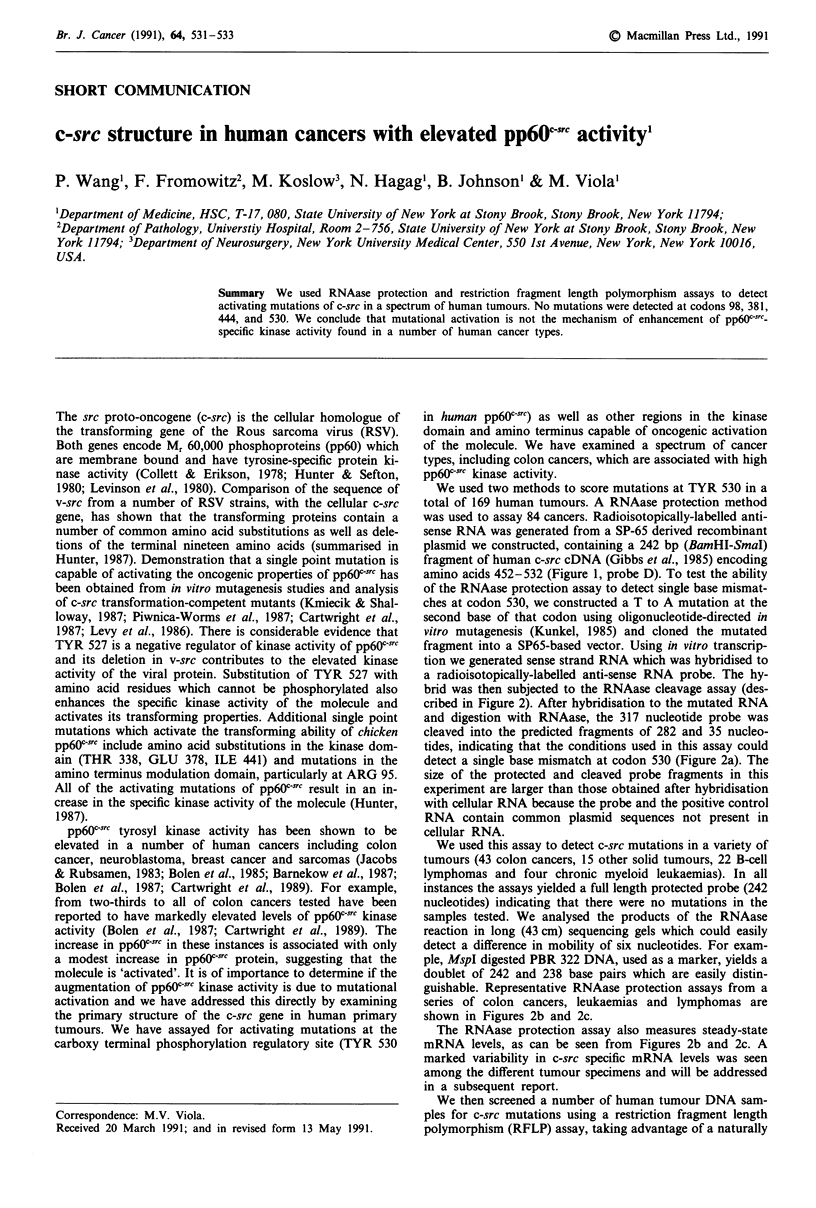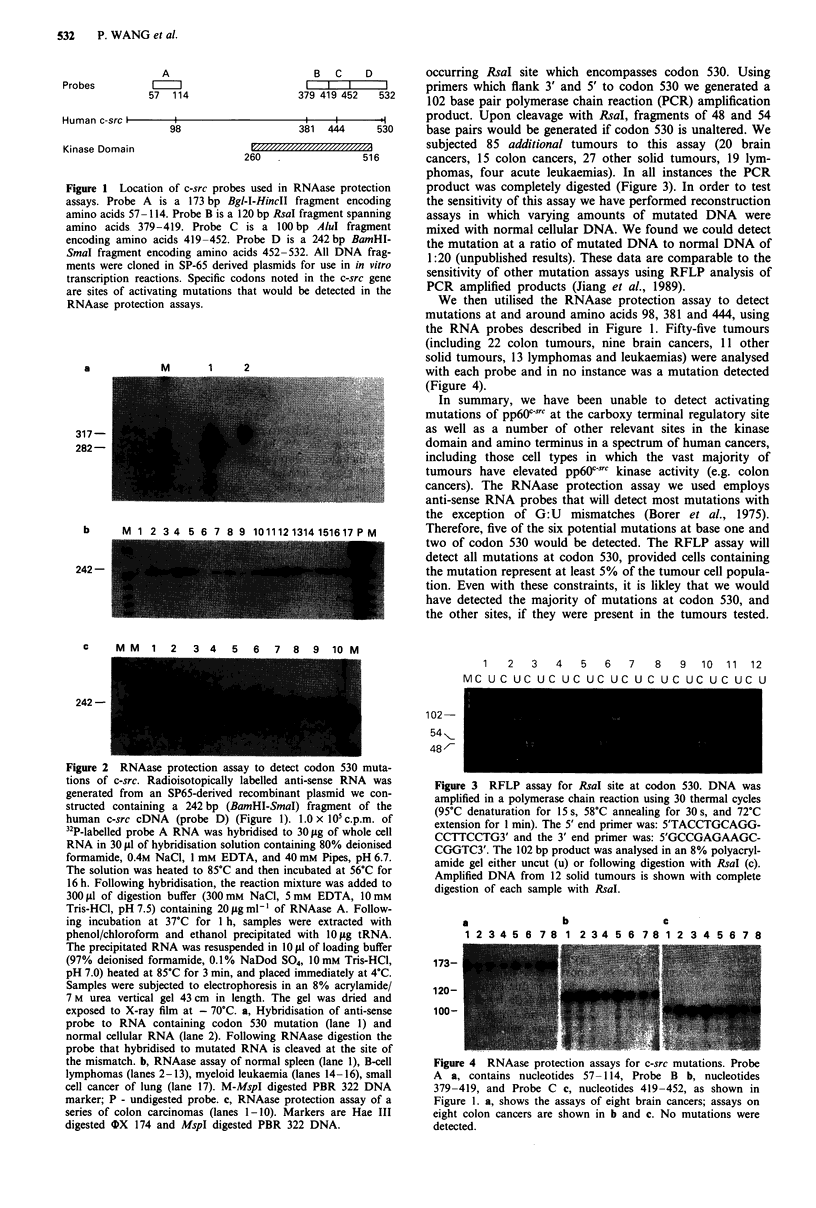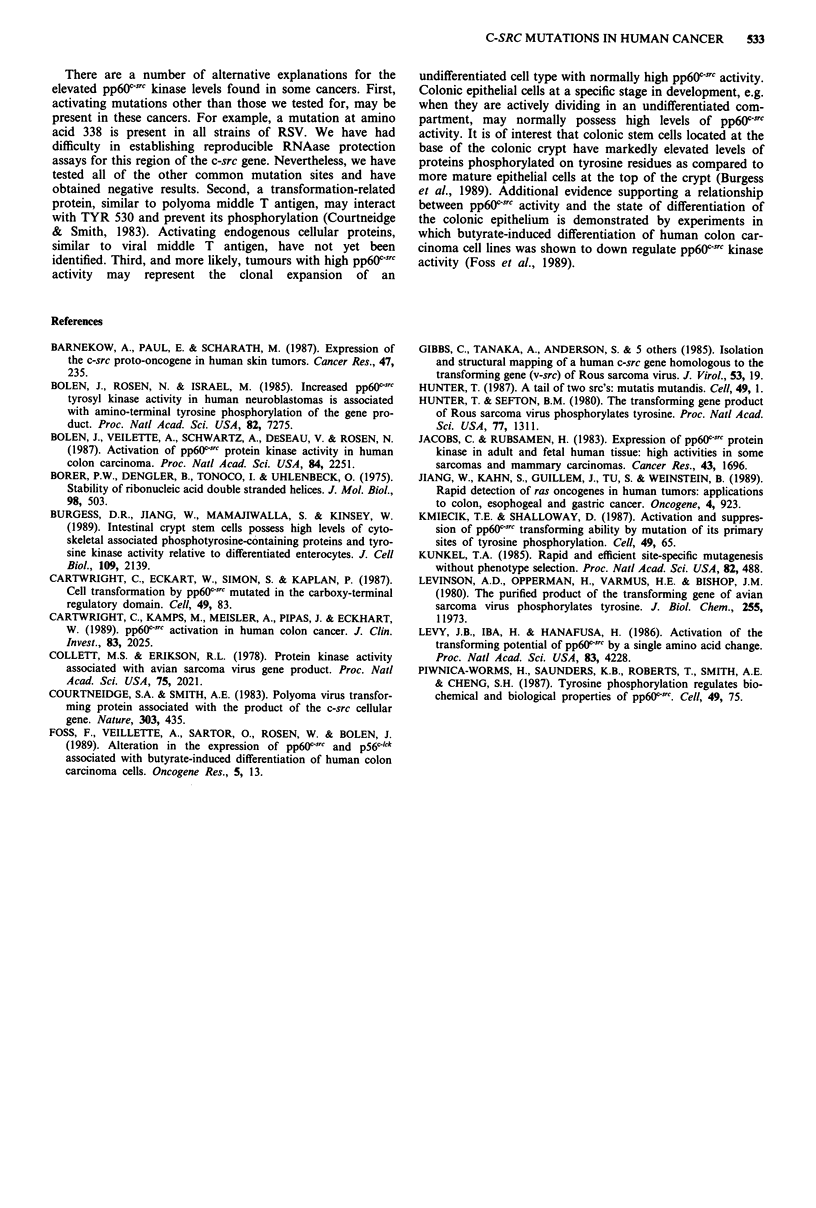# c-src structure in human cancers with elevated pp60c-src activity.

**DOI:** 10.1038/bjc.1991.344

**Published:** 1991-09

**Authors:** P. Wang, F. Fromowitz, M. Koslow, N. Hagag, B. Johnson, M. Viola

**Affiliations:** Department of Medicine, State University of New York, Stony Brook 11794.

## Abstract

**Images:**


Br. J. Cancer (1991), 64, 531–533 © Macmillan Press Ltd., 1991

SHORT COMMUNICATION

### c-*src* structure in human cancers with elevated pp60$^{c\text{-}src}$ activity[1]

P. Wang[1], F. Fromowitz[2], M. Koslow[3], N. Hagag[1], B. Johnson[1] & M. Viola[1]

[1]*Department of Medicine, HSC, T-17, 080, State University of New York at Stony Brook, Stony Brook, New York 11794;* [2]*Department of Pathology, Universtiy Hospital, Room 2–756, State University of New York at Stony Brook, Stony Brook, New York 11794;* [3]*Department of Neurosurgery, New York University Medical Center, 550 1st Avenue, New York, New York 10016, USA.*

**Summary** We used RNAase protection and restriction fragment length polymorphism assays to detect activating mutations of c-*src* in a spectrum of human tumours. No mutations were detected at codons 98, 381, 444, and 530. We conclude that mutational activation is not the mechanism of enhancement of pp60$^{c\text{-}src}$-specific kinase activity found in a number of human cancer types.

The *src* proto-oncogene (c-*src*) is the cellular homologue of the transforming gene of the Rous sarcoma virus (RSV). Both genes encode $M_r$ 60,000 phosphoproteins (pp60) which are membrane bound and have tyrosine-specific protein kinase activity (Collett & Erikson, 1978; Hunter & Sefton, 1980; Levinson *et al.*, 1980). Comparison of the sequence of v-*src* from a number of RSV strains, with the cellular c-*src* gene, has shown that the transforming proteins contain a number of common amino acid substitutions as well as deletions of the terminal nineteen amino acids (summarised in Hunter, 1987). Demonstration that a single point mutation is capable of activating the oncogenic properties of pp60$^{c\text{-}src}$ has been obtained from *in vitro* mutagenesis studies and analysis of c-*src* transformation-competent mutants (Kmiecik & Shalloway, 1987; Piwnica-Worms *et al.*, 1987; Cartwright *et al.*, 1987; Levy *et al.*, 1986). There is considerable evidence that TYR 527 is a negative regulator of kinase activity of pp60$^{c\text{-}src}$ and its deletion in v-*src* contributes to the elevated kinase activity of the viral protein. Substitution of TYR 527 with amino acid residues which cannot be phosphorylated also enhances the specific kinase activity of the molecule and activates its transforming properties. Additional single point mutations which activate the transforming ability of *chicken* pp60$^{c\text{-}src}$ include amino acid substitutions in the kinase domain (THR 338, GLU 378, ILE 441) and mutations in the amino terminus modulation domain, particularly at ARG 95. All of the activating mutations of pp60$^{c\text{-}src}$ result in an increase in the specific kinase activity of the molecule (Hunter, 1987).

pp60$^{c\text{-}src}$ tyrosyl kinase activity has been shown to be elevated in a number of human cancers including colon cancer, neuroblastoma, breast cancer and sarcomas (Jacobs & Rubsamen, 1983; Bolen *et al.*, 1985; Barnekow *et al.*, 1987; Bolen *et al.*, 1987; Cartwright *et al.*, 1989). For example, from two-thirds to all of colon cancers tested have been reported to have markedly elevated levels of pp60$^{c\text{-}src}$ kinase activity (Bolen *et al.*, 1987; Cartwright *et al.*, 1989). The increase in pp60$^{c\text{-}src}$ in these instances is associated with only a modest increase in pp60$^{c\text{-}src}$ protein, suggesting that the molecule is 'activated'. It is of importance to determine if the augmentation of pp60$^{c\text{-}src}$ kinase activity is due to mutational activation and we have addressed this directly by examining the primary structure of the c-*src* gene in human primary tumours. We have assayed for activating mutations at the carboxy terminal phosphorylation regulatory site (TYR 530 in *human* pp60$^{c\text{-}src}$) as well as other regions in the kinase domain and amino terminus capable of oncogenic activation of the molecule. We have examined a spectrum of cancer types, including colon cancers, which are associated with high pp60$^{c\text{-}src}$ kinase activity.

We used two methods to score mutations at TYR 530 in a total of 169 human tumours. A RNAase protection method was used to assay 84 cancers. Radioisotopically-labelled antisense RNA was generated from a SP-65 derived recombinant plasmid we constructed, containing a 242 bp (*Bam*HI-*Sma*I) fragment of human c-*src* cDNA (Gibbs *et al.*, 1985) encoding amino acids 452–532 (Figure 1, probe D). To test the ability of the RNAase protection assay to detect single base mismatches at codon 530, we constructed a T to A mutation at the second base of that codon using oligonucleotide-directed *in vitro* mutagenesis (Kunkel, 1985) and cloned the mutated fragment into a SP65-based vector. Using *in vitro* transcription we generated sense strand RNA which was hybridised to a radioisotopically-labelled anti-sense RNA probe. The hybrid was then subjected to the RNAase cleavage assay (described in Figure 2). After hybridisation to the mutated RNA and digestion with RNAase, the 317 nucleotide probe was cleaved into the predicted fragments of 282 and 35 nucleotides, indicating that the conditions used in this assay could detect a single base mismatch at codon 530 (Figure 2a). The size of the protected and cleaved probe fragments in this experiment are larger than those obtained after hybridisation with cellular RNA because the probe and the positive control RNA contain common plasmid sequences not present in cellular RNA.

We used this assay to detect c-*src* mutations in a variety of tumours (43 colon cancers, 15 other solid tumours, 22 B-cell lymphomas and four chronic myeloid leukaemias). In all instances the assays yielded a full length protected probe (242 nucleotides) indicating that there were no mutations in the samples tested. We analysed the products of the RNAase reaction in long (43 cm) sequencing gels which could easily detect a difference in mobility of six nucleotides. For example, *Msp*I digested PBR 322 DNA, used as a marker, yields a doublet of 242 and 238 base pairs which are easily distinguishable. Representative RNAase protection assays from a series of colon cancers, leukaemias and lymphomas are shown in Figures 2b and 2c.

The RNAase protection assay also measures steady-state mRNA levels, as can be seen from Figures 2b and 2c. A marked variability in c-*src* specific mRNA levels was seen among the different tumour specimens and will be addressed in a subsequent report.

We then screened a number of human tumour DNA samples for c-*src* mutations using a restriction fragment length polymorphism (RFLP) assay, taking advantage of a naturally

Correspondence: M.V. Viola.
Received 20 March 1991; and in revised form 13 May 1991.

**Figure 1** Location of c-*src* probes used in RNAase protection assays. Probe A is a 173 bp *Bgl*-I-*Hinc*II fragment encoding amino acids 57–114. Probe B is a 120 bp *Rsa*I fragment spanning amino acids 379–419. Probe C is a 100 bp *Alu*I fragment encoding amino acids 419–452. Probe D is a 242 bp *Bam*HI-*Sma*I fragment encoding amino acids 452–532. All DNA fragments were cloned in SP-65 derived plasmids for use in *in vitro* transcription reactions. Specific codons noted in the c-*src* gene are sites of activating mutations that would be detected in the RNAase protection assays.

**Figure 2** RNAase protection assay to detect codon 530 mutations of c-*src*. Radioisotopically labelled anti-sense RNA was generated from an SP65-derived recombinant plasmid we constructed containing a 242 bp (*Bam*HI-*Sma*I) fragment of the human c-*src* cDNA (probe D) (Figure 1). $1.0 \times 10^5$ c.p.m. of $^{32}$P-labelled probe A RNA was hybridised to 30 µg of whole cell RNA in 30 µl of hybridisation solution containing 80% deionised formamide, 0.4 M NaCl, 1 mM EDTA, and 40 mM Pipes, pH 6.7. The solution was heated to 85°C and then incubated at 56°C for 16 h. Following hybridisation, the reaction mixture was added to 300 µl of digestion buffer (300 mM NaCl, 5 mM EDTA, 10 mM Tris-HCl, pH 7.5) containing 20 µg ml$^{-1}$ of RNAase A. Following incubation at 37°C for 1 h, samples were extracted with phenol/chloroform and ethanol precipitated with 10 µg tRNA. The precipitated RNA was resuspended in 10 µl of loading buffer (97% deionised formamide, 0.1% NaDod $SO_4$, 10 mM Tris-HCl, pH 7.0) heated at 85°C for 3 min, and placed immediately at 4°C. Samples were subjected to electrophoresis in an 8% acrylamide/7 M urea vertical gel 43 cm in length. The gel was dried and exposed to X-ray film at −70°C. **a**, Hybridisation of anti-sense probe to RNA containing codon 530 mutation (lane 1) and normal cellular RNA (lane 2). Following RNAase digestion the probe that hybridised to mutated RNA is cleaved at the site of the mismatch. **b**, RNAase assay of normal spleen (lane 1), B-cell lymphomas (lanes 2–13), myeloid leukaemia (lanes 14–16), small cell cancer of lung (lane 17). M-*Msp*I digested PBR 322 DNA marker; P - undigested probe. **c**, RNAase protection assay of a series of colon carcinomas (lanes 1–10). Markers are Hae III digested ΦX 174 and *Msp*I digested PBR 322 DNA.

occurring *Rsa*I site which encompasses codon 530. Using primers which flank 3′ and 5′ to codon 530 we generated a 102 base pair polymerase chain reaction (PCR) amplification product. Upon cleavage with *Rsa*I, fragments of 48 and 54 base pairs would be generated if codon 530 is unaltered. We subjected 85 *additional* tumours to this assay (20 brain cancers, 15 colon cancers, 27 other solid tumours, 19 lymphomas, four acute leukaemias). In all instances the PCR product was completely digested (Figure 3). In order to test the sensitivity of this assay we have performed reconstruction assays in which varying amounts of mutated DNA were mixed with normal cellular DNA. We found we could detect the mutation at a ratio of mutated DNA to normal DNA of 1:20 (unpublished results). These data are comparable to the sensitivity of other mutation assays using RFLP analysis of PCR amplified products (Jiang *et al.*, 1989).

We then utilised the RNAase protection assay to detect mutations at and around amino acids 98, 381 and 444, using the RNA probes described in Figure 1. Fifty-five tumours (including 22 colon tumours, nine brain cancers, 11 other solid tumours, 13 lymphomas and leukaemias) were analysed with each probe and in no instance was a mutation detected (Figure 4).

In summary, we have been unable to detect activating mutations of pp60$^{c\text{-}src}$ at the carboxy terminal regulatory site as well as a number of other relevant sites in the kinase domain and amino terminus in a spectrum of human cancers, including those cell types in which the vast majority of tumours have elevated pp60$^{c\text{-}src}$ kinase activity (e.g. colon cancers). The RNAase protection assay we used employs anti-sense RNA probes that will detect most mutations with the exception of G:U mismatches (Borer *et al.*, 1975). Therefore, five of the six potential mutations at base one and two of codon 530 would be detected. The RFLP assay will detect all mutations at codon 530, provided cells containing the mutation represent at least 5% of the tumour cell population. Even with these constraints, it is likley that we would have detected the majority of mutations at codon 530, and the other sites, if they were present in the tumours tested.

**Figure 3** RFLP assay for *Rsa*I site at codon 530. DNA was amplified in a polymerase chain reaction using 30 thermal cycles (95°C denaturation for 15 s, 58°C annealing for 30 s, and 72°C extension for 1 min). The 5′ end primer was: 5′TACCTGCAGG-CCTTCCTG3′ and the 3′ end primer was: 5′GCCGAGAAGC-CGGTC3′. The 102 bp product was analysed in an 8% polyacrylamide gel either uncut (u) or following digestion with *Rsa*I (c). Amplified DNA from 12 solid tumours is shown with complete digestion of each sample with *Rsa*I.

**Figure 4** RNAase protection assays for c-*src* mutations. Probe A **a**, contains nucleotides 57–114, Probe B **b**, nucleotides 379–419, and Probe C **c**, nucleotides 419–452, as shown in Figure 1. **a**, shows the assays of eight brain cancers; assays on eight colon cancers are shown in **b** and **c**. No mutations were detected.

There are a number of alternative explanations for the elevated pp60$^{c\text{-}src}$ kinase levels found in some cancers. First, activating mutations other than those we tested for, may be present in these cancers. For example, a mutation at amino acid 338 is present in all strains of RSV. We have had difficulty in establishing reproducible RNAase protection assays for this region of the c-*src* gene. Nevertheless, we have tested all of the other common mutation sites and have obtained negative results. Second, a transformation-related protein, similar to polyoma middle T antigen, may interact with TYR 530 and prevent its phosphorylation (Courtneidge & Smith, 1983). Activating endogenous cellular proteins, similar to viral middle T antigen, have not yet been identified. Third, and more likely, tumours with high pp60$^{c\text{-}src}$ activity may represent the clonal expansion of an undifferentiated cell type with normally high pp60$^{c\text{-}src}$ activity. Colonic epithelial cells at a specific stage in development, e.g. when they are actively dividing in an undifferentiated compartment, may normally possess high levels of pp60$^{c\text{-}src}$ activity. It is of interest that colonic stem cells located at the base of the colonic crypt have markedly elevated levels of proteins phosphorylated on tyrosine residues as compared to more mature epithelial cells at the top of the crypt (Burgess *et al.*, 1989). Additional evidence supporting a relationship between pp60$^{c\text{-}src}$ activity and the state of differentiation of the colonic epithelium is demonstrated by experiments in which butyrate-induced differentiation of human colon carcinoma cell lines was shown to down regulate pp60$^{c\text{-}src}$ kinase activity (Foss *et al.*, 1989).

### References

BARNEKOW, A., PAUL, E. & SCHARATH, M. (1987). Expression of the c-*src* proto-oncogene in human skin tumors. *Cancer Res.*, **47**, 235.

BOLEN, J., ROSEN, N. & ISRAEL, M. (1985). Increased pp60$^{c\text{-}src}$ tyrosyl kinase activity in human neuroblastomas is associated with amino-terminal tyrosine phosphorylation of the gene product. *Proc. Natl Acad. Sci. USA*, **82**, 7275.

BOLEN, J., VEILETTE, A., SCHWARTZ, A., DESEAU, V. & ROSEN, N. (1987). Activation of pp60$^{c\text{-}src}$ protein kinase activity in human colon carcinoma. *Proc. Natl Acad. Sci. USA*, **84**, 2251.

BORER, P.W., DENGLER, B., TONOCO, I. & UHLENBECK, O. (1975). Stability of ribonucleic acid double stranded helices. *J. Mol. Biol.*, **98**, 503.

BURGESS, D.R., JIANG, W., MAMAJIWALLA, S. & KINSEY, W. (1989). Intestinal crypt stem cells possess high levels of cytoskeletal associated phosphotyrosine-containing proteins and tyrosine kinase activity relative to differentiated enterocytes. *J. Cell Biol.*, **109**, 2139.

CARTWRIGHT, C., ECKART, W., SIMON, S. & KAPLAN, P. (1987). Cell transformation by pp60$^{c\text{-}src}$ mutated in the carboxy-terminal regulatory domain. *Cell*, **49**, 83.

CARTWRIGHT, C., KAMPS, M., MEISLER, A., PIPAS, J. & ECKHART, W. (1989). pp60$^{c\text{-}src}$ activation in human colon cancer. *J. Clin. Invest.*, **83**, 2025.

COLLETT, M.S. & ERIKSON, R.L. (1978). Protein kinase activity associated with avian sarcoma virus gene product. *Proc. Natl Acad. Sci. USA*, **75**, 2021.

COURTNEIDGE, S.A. & SMITH, A.E. (1983). Polyoma virus transforming protein associated with the product of the c-*src* cellular gene. *Nature*, **303**, 435.

FOSS, F., VEILLETTE, A., SARTOR, O., ROSEN, W. & BOLEN, J. (1989). Alteration in the expression of pp60$^{c\text{-}src}$ and p56$^{c\text{-}lck}$ associated with butyrate-induced differentiation of human colon carcinoma cells. *Oncogene Res.*, **5**, 13.

GIBBS, C., TANAKA, A., ANDERSON, S. & 5 others (1985). Isolation and structural mapping of a human c-*src* gene homologous to the transforming gene (v-*src*) of Rous sarcoma virus. *J. Virol.*, **53**, 19.

HUNTER, T. (1987). A tail of two src's: mutatis mutandis. *Cell*, **49**, 1.

HUNTER, T. & SEFTON, B.M. (1980). The transforming gene product of Rous sarcoma virus phosphorylates tyrosine. *Proc. Natl Acad. Sci. USA*, **77**, 1311.

JACOBS, C. & RUBSAMEN, H. (1983). Expression of pp60$^{c\text{-}src}$ protein kinase in adult and fetal human tissue: high activities in some sarcomas and mammary carcinomas. *Cancer Res.*, **43**, 1696.

JIANG, W., KAHN, S., GUILLEM, J., TU, S. & WEINSTEIN, B. (1989). Rapid detection of *ras* oncogenes in human tumors: applications to colon, esophogeal and gastric cancer. *Oncogene*, **4**, 923.

KMIECIK, T.E. & SHALLOWAY, D. (1987). Activation and suppression of pp60$^{c\text{-}src}$ transforming ability by mutation of its primary sites of tyrosine phosphorylation. *Cell*, **49**, 65.

KUNKEL, T.A. (1985). Rapid and efficient site-specific mutagenesis without phenotype selection. *Proc. Natl Acad. Sci. USA*, **82**, 488.

LEVINSON, A.D., OPPERMAN, H., VARMUS, H.E. & BISHOP, J.M. (1980). The purified product of the transforming gene of avian sarcoma virus phosphorylates tyrosine. *J. Biol. Chem.*, **255**, 11973.

LEVY, J.B., IBA, H. & HANAFUSA, H. (1986). Activation of the transforming potential of pp60$^{c\text{-}src}$ by a single amino acid change. *Proc. Natl Acad. Sci. USA*, **83**, 4228.

PIWNICA-WORMS, H., SAUNDERS, K.B., ROBERTS, T., SMITH, A.E. & CHENG, S.H. (1987). Tyrosine phosphorylation regulates biochemical and biological properties of pp60$^{c\text{-}src}$. *Cell*, **49**, 75.